# Pyrimidine addiction: an Achilles’ heel of NF2-altered mesothelioma

**DOI:** 10.1038/s44321-025-00276-6

**Published:** 2025-07-24

**Authors:** Jianlong Jia, Georgios T Stathopoulos

**Affiliations:** 1 Comprehensive Pneumology Center (CPC), Institute of Lung Health and Immunity (LHI), Helmholtz Munich, Member of the German Center for Lung Research (DZL), Munich, Germany; 2 Ludwig-Maximilians-University (LMU), Munich, Germany; 3 Medical School, University of Cyprus, Aglantzia, Nicosia, Cyprus

**Keywords:** Cancer, Metabolism, Respiratory System

## Abstract

In this N&V, G. Stathopoulos and J. Jia discuss the article by Xu et al, in the current issue of EMBO Molecular Medicine, that identifies de novo pyrimidine synthesis as a synthetic lethal vulnerability in NF2-deficient pleural mesothelioma.

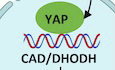

## Teaser

Therapeutic options for patients with mesothelioma remain scarce and five-year survival is a meager ten percent. According to data from three large studies, 36% of mesotheliomas harbor neurofibromatosis 2 (NF2) mutations or loss (https://bit.ly/4nGcRXk; accessed on 06.30.2025), yet no targeted therapy exists for this molecular subtype of the disease. In the current issue of *EMBO Molecular Medicine*, Xu et al, demonstrate that NF2-deficiency of mesothelioma cells releases an NF2-mediated Hippo-pathway restraint, activates the Yes-associated protein (YAP)–carbamoyl-phosphate synthetase 2, aspartate transcarbamylase, and dihydroorotase (CAD)/dihydroorotate dehydrogenase (DHODH) axis, and drives addiction to high-flux de novo pyrimidine biosynthesis (Xu et al, 2025). Moreover, the authors elegantly show that DHODH inhibitors elicit robust antitumor activity against NF2-altered mesothelioma and exhibit strong synergy with cisplatin, opening a new translational avenue for this tumor subtype.

**Figure 1:**
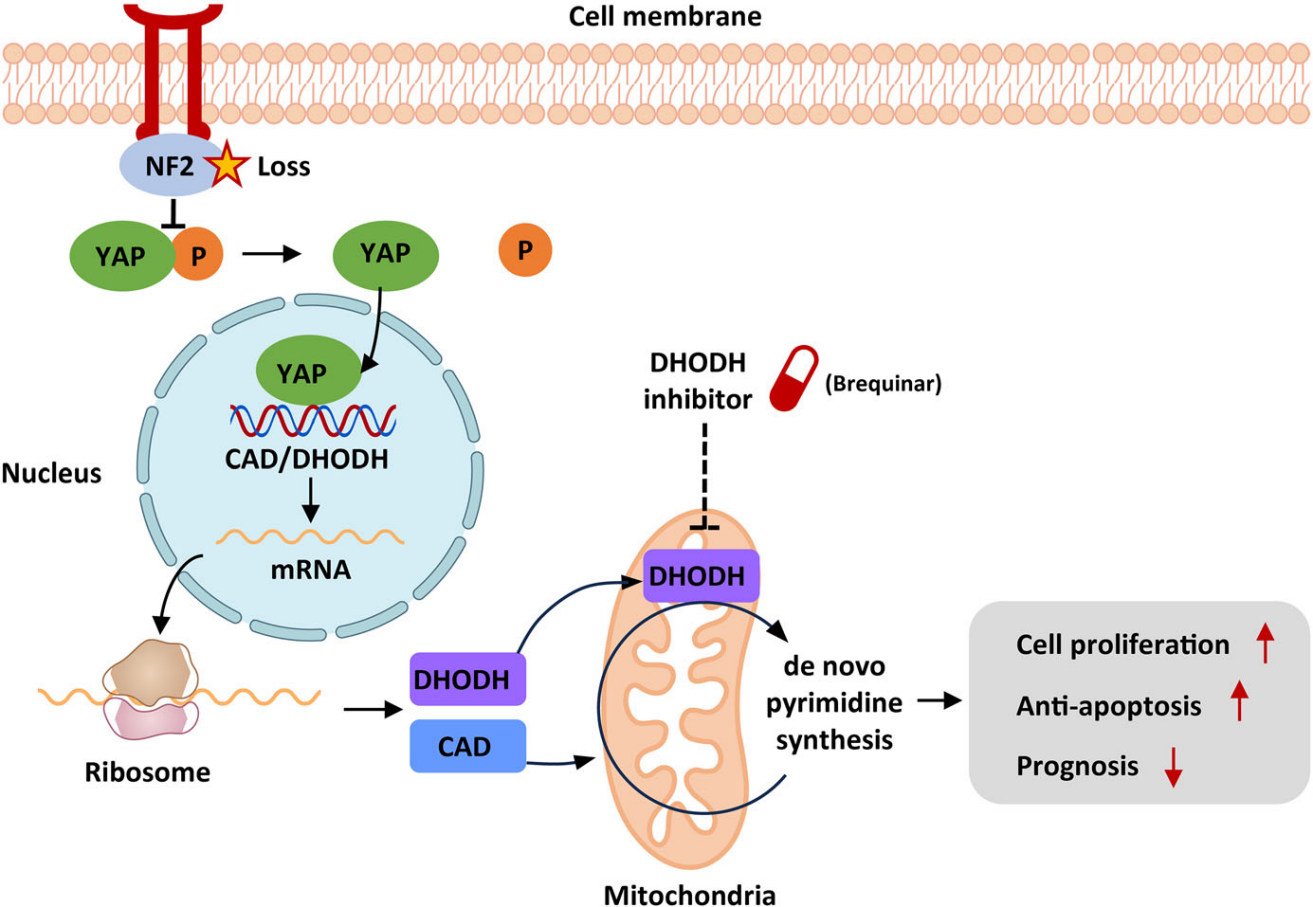
NF2 loss unleashes a metabolic pyrimidine addiction that can be pharmacologically exploited. NF2 deficiency eliminates LATS-mediated phosphorylation of YAP, allowing dephosphorylated YAP to enter the nucleus and co-activate CAD and DHODH transcription. Together, these enzymes accelerate de novo pyrimidine synthesis, enlarge the cellular UMP pool, and thereby fuel rapid proliferation while resisting apoptosis. The pathway’s bottleneck, DHODH, can be selectively blocked by inhibitors such as Brequinar, revealing a precision-metabolic vulnerability in NF2-deficient mesothelioma. Figure partly generated using Biovisart (https://biovisart.com.cn).

Mesothelioma is mainly caused by occupational asbestos exposure, and bears therefore important socioeconomic implications and an urgent need for cure (Carbone et al, 2019). Yet surgery only benefits a minority of patients, and the objective response rates to platinum chemotherapy and dual immune checkpoint inhibition (nivolumab plus ipilimumab) is less than 40% (Janes et al, 2021). To make matters worse, the molecular landscape of mesothelioma is mostly dominated by inactivated tumor suppressor genes (*NF2*, *BAP1*, *TP53*, *CDKN2A/2B*, etc.) rather than druggable driver genes (Bueno et al, 2016), rendering targeted therapy impossible. Faced with this conundrum, Xu et al turned to synthetic lethality: using the metabolic dependence caused by NF2 loss to deprive mesothelioma cells of a critical metabolic fuel. Pyrimidine synthesis has long been regarded as a druggable node, and the DHODH inhibitor leflunomide has already entered the clinic (Eckhardt et al, 1999). However, its specificity against specific tumor genotypes is unclear. Xu et al deliver three pioneering advances: First, they ascribe a pyrimidine‑enriched transcriptome specifically to NF2-altered mesothelioma; second, they demonstrate that YAP directly binds the CAD promoter and a DHODH enhancer, markedly increasing de novo pyrimidine biosynthesis pathway flux and creating a vulnerability to DHODH inhibitors; lastly, they demonstrate potent in vivo synergy of such compounds with cisplatin, lending hope for possible future integration of pyrimidine pathway blockade into current treatment regimens (Fig. 1).

In addition to the above, Xu et al also provide mechanistic advances. They employed state-of-the-art multi-omics integrating transcriptome, proteome, and metabolome datasets in order to interrogate the metabolic heterogeneity of mesothelioma. Strikingly, they report that NF2 deficiency delineates a distinct molecular subtype marked by pronounced up-regulation of de novo pyrimidine biosynthesis, a metabolic pathway indispensable for supplying nucleotides required for DNA replication and repair (Bajzikova et al, 2019). To this end, it is shown that NF2 loss-of-function leads to large tumor suppressor kinases (LATS) 1 and 2 inactivation and consequent dephosphorylation of YAP at Ser127, allowing its nuclear translocation along the canonical Hippo pathway. ChIP-qPCR and dual luciferase reporter assays are further employed to discover that dephosphorylated YAP binds directly to the CAD promoter and a distal enhancer of DHODH. CAD is the catalytic core of the trifunctional enzyme that drives the initial steps of de novo pyrimidine synthesis. DHODH, an inner mitochondrial membrane oxidoreductase, converts dihydroorotate to orotate while transferring electrons to coenzyme Q. Coordinated up-regulation of CAD and DHODH doubles pyrimidine pathway flux, as revealed by 15^N^-glutamine tracing and Uridine-5′-monophosphate (UMP) labeling. This metabolic rewiring is not merely epiphenomenal; it represents a critical dependency for the proliferation and survival of NF2-deficient mesothelioma cells. Extensive in vitro experiments demonstrate that genetic silencing or pharmacological inhibition of CAD or DHODH markedly compromises cell viability, growth, and proliferation, specifically in the NF2-deficient setting. Together, these layers of evidence pinpoint de novo pyrimidine biogenesis as an Achilles’ heel of NF2-deficient mesothelioma.

Intriguingly, whereas normal or slowly proliferating cells rely mainly on nucleoside salvage, NF2-deficient cells exhibit exquisite vulnerability to DHODH blockade under standard nutrient conditions, a liability that persists even after glucose restriction but can be fully rescued by high exogenous uridine. These results indicate that NF2-altered tumors stake their survival on de novo pyrimidine synthesis. Such extreme reliance has been documented only in oncogene-driven tumors, e.g., *KRAS*-mutant lung cancer or *MYC*-amplified brain tumors (Gwynne et al, 2022; Koundinya et al, 2018), and Xu et al extend this concept to NF2-altered mesothelioma. Validation across rigorous preclinical models confirms the therapeutic promise of DHODH targeting and highlights a potent combination strategy: DHODH inhibition specifically synergizes with cisplatin against NF2-deficient mesothelioma, enhancing antitumor efficacy while limiting potential resistance pathways and underscoring the regimen’s robust translational and clinical potential. We believe that such regimens should also be tested against *KRAS*-altered mesotheliomas, a molecular subclass we recently identified (Marazioti et al, 2022).

According to Xu et al, roughly half of mesotheliomas display a pyrimidine-high phenotype driven by NF2 deficiency, high CAD/DHODH expression, and the poorest prognosis, most likely the population to benefit the most from targeting de novo pyrimidine synthesis. Luckily, there are multiple options for this to be achieved. Leflunomide, an oral DHODH inhibitor with an extensive safety record is already available (Eckhardt et al, 1999). Brequinar, another DHODH inhibitor, has not been successful clinically, however, early studies dosed the drug in molecularly unselected cohorts, raising the possibility that any genuine efficacy signal was masked by background noise (Cody et al, 1993). It is conceivable that brequinar could display improved safety and efficacy when specifically deployed against NF2-altered mesothelioma.

In conclusion, the work by Xu et al published in the current issue of *EMBO Molecular Medicine* delineates a druggable metabolic addiction of NF2-deficient mesothelioma to pyrimidine and offers a novel translational opportunity to treat this otherwise intractable neoplasm. This work underscores the transformative potential of precision metabolic interventions in personalized oncology.
